# Percutaneous Navigation under Local Anesthesia for Computed Tomography-Guided Microwave Ablation of Malignant Liver Lesions Located in the Hepatic Dome

**DOI:** 10.3390/medicina57101056

**Published:** 2021-10-03

**Authors:** Dimitrios K. Filippiadis, Georgios Velonakis, Argyro Mazioti, Athanasios Tsochatzis, Thomas Vrachliotis, Alexis Kelekis, Nikolaos Kelekis

**Affiliations:** 2nd Department of Radiology, University General Hospital “ATTIKON”, Medical School, National and Kapodistrian University of Athens, 1 Rimini Str., 12462 Athens, Greece; giorvelonakis@gmail.com (G.V.); argyromazioti@yahoo.gr (A.M.); thanasis.tsochatzis@hotmail.com (A.T.); vrachliotis2@yahoo.com (T.V.); akelekis@med.uoa.gr (A.K.); kelnik@med.uoa.gr (N.K.)

**Keywords:** microwave ablation, navigation, computed tomography, local anesthesia, hepatocellular carcinoma, metastasis

## Abstract

*Background and Objectives:* The aim of the present study was to report the safety and efficacy of percutaneous navigation under local anesthesia for computed tomography-guided microwave ablation of malignant liver lesions located in the hepatic dome. Patients with primary and secondary malignant liver lesions located in the hepatic dome who underwent percutaneous computed tomography-guided microwave ablation using a computer-assisted navigation system under local anesthesia were prospectively evaluated. The primary objective was technical success. *Materials and Methods:* The sample consisted of 10 participants (16 lesions) with a mean age of 60.60 years (SD = 9.25 years) and a mean size of 20.37 ± 7.29 cm, and the mean follow-up time was 3.4 months (SD = 1.41) months. *Results:* Primary technical success was 93.75%. Tumor remnant was noticed at one month follow-up in a single metastatic lesion, which was re-treated with an ablation session, and no tumor remnant was depicted in the subsequent imaging follow-up (secondary technical success 100%). Grade I self-limited complications (according to the CIRSE classification system) included small pleural effusion (*n* = 1) and minor bleeding post antenna removal (*n* = 1) requiring nothing but observation. *Conclusions:* the findings of the present study indicate that percutaneous navigation under local anesthesia is a safe and efficacious approach for computed tomography-guided microwave ablation of malignant liver lesions located in the hepatic dome. Large randomized controlled studies are warranted to observe treatment effectiveness and compare the results with those of other options.

## 1. Introduction

Percutaneous ablation of primary and secondary liver lesions constitutes a well-established therapeutic technique for the management of hepatocellular carcinoma or hepatic metastases of various neoplasmatic origin [1,2,3]. Almost all diagnostic methods have been used either solely or in combination (with or without fusion imaging) for ablation guidance [4]. In the vast majority of ablation sessions, a conventional manual free-hand approach is performed; more recently, however, a stereotactic computer-assisted navigation has been applied, aiming to improve the precision of the needle placement [5,6,7,8,9,10]. The CT-navigation system allows for real-time treatment planning during the procedure and helps to avoid critical structures without angular limitations for an optimal trajectory [11]. General anesthesia or deep sedation seems to be a prerequisite for the application of this stereotactic, computer-assisted navigation [5,6,7,8,9,10,11]. Unfortunately, although anesthesiologists are best trained, they are not available to attend all ablation procedures [12].

The anatomical location of a hepatic tumor significantly affects local recurrence post percutaneous ablation [13]. Challenging locations for tumor ablation in the liver include, among others, the hepatic dome, areas close to the liver hilum or to the heart, and subcapsular locations [14]. In the hepatic dome, apart from the approach of a needle which is angulated in the vast majority of the cases (increasing the difficulty level), the presence of the diaphragm results in a shift towards a conservative ablation strategy. Although this serves as an attempt to avoid complications from either direct penetration or more commonly thermal trauma, it can result in under-treatment [15].

The aim of the present study was to report the safety and efficacy of percutaneous navigation under local anesthesia for computed tomography-guided microwave ablation of malignant liver lesions located in the hepatic dome.

## 2. Materials and Methods

### 2.1. Patient Selection and Evaluation

The present study is a prospective observational study evaluating patients with primary and secondary malignant liver lesions located in the hepatic dome; all lesions were treated with percutaneous computed tomography-guided microwave ablation using a computer-assisted navigation system under local anesthesia. The primary objective was technical success. Secondary objectives included an evaluation of complications. All included lesions were evaluable for the 3-month follow-up. Inclusion criteria included patients ≥18 years old with primary and secondary malignant liver lesions located in the hepatic dome, coagulation parameters within normal limits, and a life expectancy of >3 months. Exclusion criteria included non-compliance of patients, uncontrollable INR, systematic or local infection, expected survival less than 3 months, ECOG score less than 3, and presence of a medical or psychiatric illness that would preclude informed consent or follow-up. Each patient underwent laboratory tests at least 24 h prior to the percutaneous ablation session. The patients were fully informed about the procedure, the possible complications, and the surgical alternatives available; informed written consent for both the technique and the study was obtained in all cases. Indication for microwave ablation was determined at a multidisciplinary tumor board. Patient characteristics, ablation and navigation technique, efficacy, and complications were evaluated.

### 2.2. Percutaneous Computed Tomography-Guided Microwave Ablation Using a Stereotactic, Computer-Assisted Navigation System

According to directions provided by the Infection Division of Pathology Department, prophylactic antibiotic was intravenously administered 45–60 min before MWA session and repeated twice over 24 h. A single operator with 12 years of experience performed all ablation sessions; the operator’s experience using the percutaneous computer-assisted navigation system was only 3 months. Microwave ablation was always performed in an inpatient setting under local anesthesia (10 cc of 2% Lidocaine Hydrochloric on skin and subcutaneous tissues) and intravenous analgesia (1 gr paracetamol and 100 mg of tramadol diluted in 100 mL of normal saline were administered during the procedure). Under local sterility, microwave ablation was performed with the percutaneous approach in all lesions.

**Figure 1:**
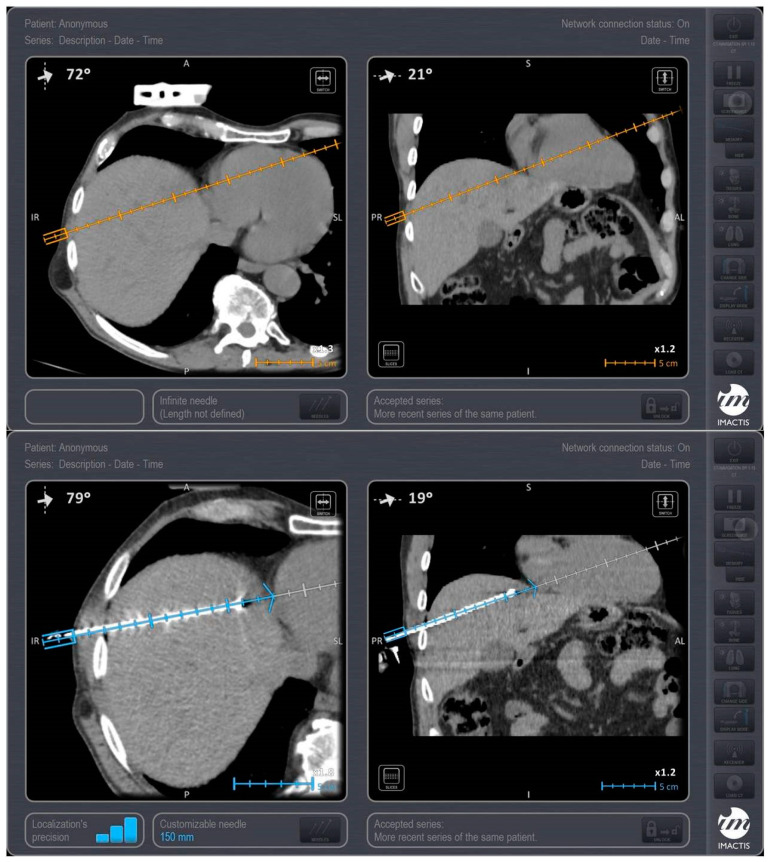
A 65-year-old male patient with colorectal cancer located at left colon with 2 synchronous metastatic lesions at hepatic dome (segment VIII). Stereotactic CT-guided ablation post systemic chemotherapy, and prior to colon resection was performed with the patient in supine position, through the anterolateral approach, under iv analgesia. Upper row: Planning scan for designing the trajectory of approach (orange line) using the IMACTIS CT navigation system. Lower row: Control scan evaluating the antenna’s position in the target lesion using the IMACTIS CT navigation system.

**Figure 2:**
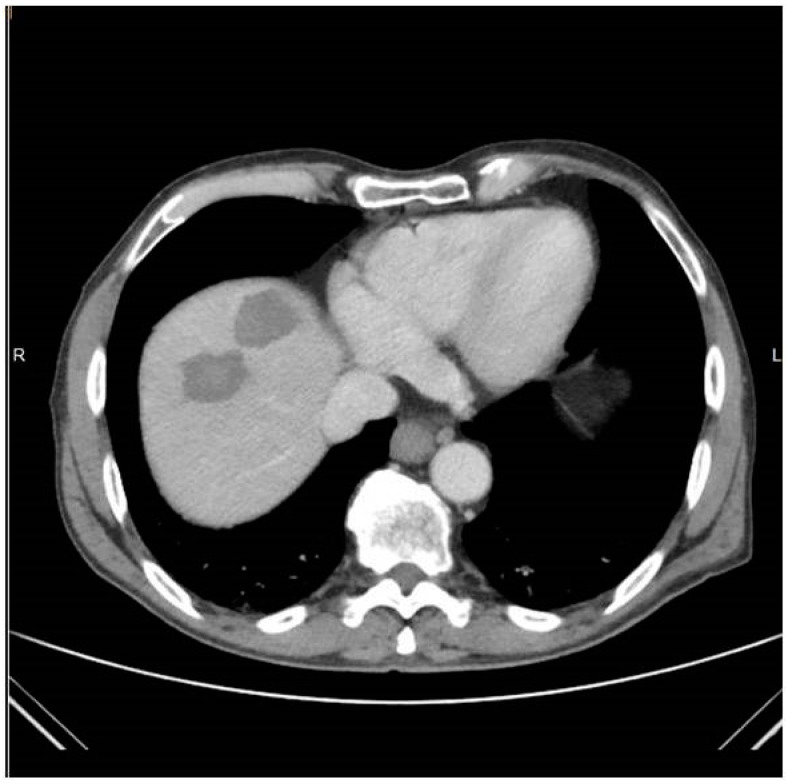
Same patient as in Figure 1: Computed tomography axial scan post intravenous contrast medium injection in the portal venous phase immediately post ablation evaluating the ablation zone and desired safety margins.

Trajectory planning and insertion of the microwave antenna were conducted using a commercially available navigation system for interventional radiology (IMACTIS SAS, Saint Martin d’Hères, France), (Figure 1). Educating the patient for correct breathing is of outmost importance; in the present study, all scans (both set-up and control ones) as well as needle movements were performed in the end-expiration apnea. In all lesions included in the present study, ablation was performed using a single microwave antenna. Once in the correct location, the ablation session was set up and performed according to the coagulation charts provided by the manufacturer in consideration of the tumor size and location and the desired safety margin. In all sessions, track ablation was performed during antenna removal from the liver in order to reduce potential risk of bleeding and peritoneal tumor seeding. CT scan in the arterial and portal venous phases validated the ablation zone and evaluated any potential immediate complications at the end of the MWA treatment (Figure 2). All patients were hospitalized overnight.

### 2.3. Outcome Measures

Technical success was defined as successful completion of the planned microwave ablation of each target lesion. Treatment outcome was reported based on standard reporting criteria [16]. Patients had a follow-up imaging with either contrast-enhanced CT or MRI at 1 and 3 months after the ablation combined with a consultation. Patients with a recurrent or residual disease were consulted for their options. Patient demographics (age, sex) as well as tumor characteristics, microwave technique, pattern of recurrence, and survival rate were evaluated. Technical success was defined as complete tumor necrosis after a single microwave ablation session with no evidence of tumor remnant or recurrence on subsequent cross-sectional imaging [17]. Progression-free survival was defined as the time interval post MWA without evidence of local recurrence. Recurrence-free survival rate was defined as the time elapsed between the intervention and any recurrence (local, regional, or distant). The definition of complications was assigned according to the Cardiovascular and Interventional Radiological Society of Europe (CIRSE) classification system [18].

### 2.4. Statistical Analysis

Patient characteristics and results were presented by means of descriptive statistics. Total number, percentage, mean and standard deviation, or median and range were used to present continuous data. Analyses were performed using acommercially available software (SPSS version 25).

## 3. Results

Demographic and clinical data of patients and lesions included in the present study are presented in Table 1. The sample consisted by 10 participants (16 lesions). The mean age of the included patients was 60.60 years (SD = 9.25 years). Neoplasmatic substrate included: hepatocellular carcinoma [2/10 (20%)], colorectal carcinoma [4/10 (40%)], gastrointestinal stromal tumor [2/10 (20%)], and myeloid carcinoma of the thyroid gland [2/10 (20%)]. The mean size of the lesions was 20.37 ± 7.29 mm, and maximum tumor size ranged from 8 to 30 mm Mean follow-up time was 3.4 months (SD = 1.41) months. Most of the participants were males, with the percentage being 80%. In the 2 HCC patients’ percutaneous ablation was decided in the multidisciplinary tumor board meeting as a first-line therapy due to co-morbidities, lesion location, and size (< 3cm in diameter). As far as the four patients with colorectal cancel liver metastases are concerned, ¾ patients involved metachronous lesions post surgery of the original intestinal tumor and systemic chemotherapy and ¼ patients suffered from synchronous hepatic lesions. In this last patient, percutaneous ablation was performed post systemic chemotherapy, whilst surgical operation of the intestinal tumor was performed the morning post ablation. In the remaining 4/10 patients, percutaneous ablation was performed in metachronous metastatic disease resistant to systemic therapies and post surgical operation of the original tumor. Technical success was 100% (i.e., antenna placement at the target lesion was successful in all patients). There was no need for hydrodissection or any other ancillary methods.

**Figure 3:**
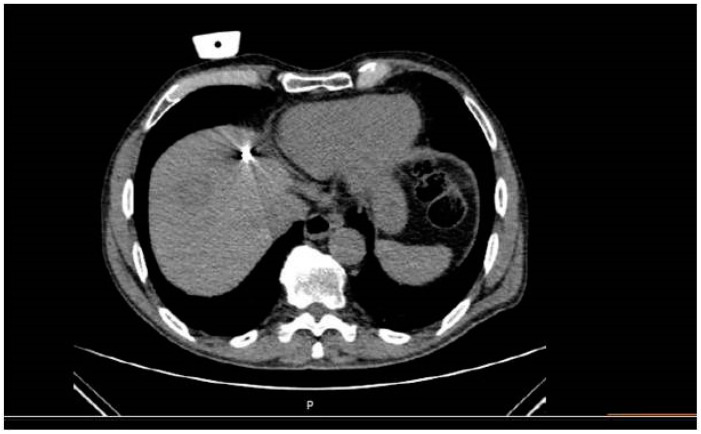
Same patient as in Figure 1 and Figure 2: Computed tomography axial scan during evaluation of the placement of the antenna in the target lesion and potential gas bubbles dispersion.

The mean total duration of the procedure from entrance to exit of the patient was 49.45 (SD = 7.53 min). Specifically, a median of 7 min was necessary for planning time and 12 min for insertion time. Whenever deemed necessary (4/16 sessions), the microwave antenna was re-positioned and a second ablation session was performed, so as to ensure that the final ablation completely encompassed both the target tumor and an annular safety zone around it that was a minimum of 5 mm thick. Navigation was used for all four repositioning. A median of 11 scans was performed including planning and control scans as well as a scan during ablation (Figure 3) and immediate imaging follow-up with 3 scans (prior to and post contrast medium injection in the arterial and portal venous phases).

On a per lesion basis, tumor remnant was noticed at one month follow-up in a single metastatic lesion (from myeloid carcinoma of the thyroid gland) (1/16, primary technical success 93.75%). This lesion was re-treated with an ablation session and no tumor remnant was depicted in the subsequent imaging follow-up (secondary technical success 100%). On a per patient basis, no disease progression was depicted in or outside the liver. Grade I self-limited complications (according to the CIRSE classification system) included small pleural effusion (*n* = 1) and active extravasation post antenna removal (*n* = 1) requiring nothing but observation. In all cases, both imaging and clinical control was performed in the first 24 h confirming the lack of a need for any further actions.

## 4. Discussion

The main problematic issue in ablation of liver tumors is incomplete ablation. Pre-requisites contributing to the prevention of incomplete ablation include the location and diameter of the tumor, proximity to vessels, accurate positioning of the ablation antenna(s) in the tumor, sufficient energy deposition, and lastly evaluation of the ablation zone. The present study adds to the growing number of case series showing that percutaneous microwave ablation under stereotactic navigation is feasible, safe, and efficacious for the treatment of malignant liver lesions [5,6,7,8,9,10,11]. Perodin et al. retrospectively evaluated 23 patients (40 liver lesions) undergoing percutaneous stereotactic imaging-guided microwave ablation, reporting an incomplete ablation rate of only 2.5% [5]. Schaible et al. retrospectively evaluated 221 patients (423 liver lesions), comparing stereotactic and conventional manual guidance. The authors concluded that percutaneous microwave ablation under stereotactic guidance exhibited significantly greater primary efficacy than conventional manual guidance [6]. Tingueli et al. investigated factors influencing the targeting accuracy and treatment efficacy of percutaneous stereotactic image-guided microwave ablation for malignant liver neoplasms, concluding that even for lesions in challenging locations, navigation allows precise and effective treatment, expanding treatment eligibility for patients with otherwise difficult to target tumors [7]. In the present study, all treated lesions were located in a challenging location (hepatic dome) and the primary technical efficacy was 93.75%, with successful ablation in 15/16 target lesions. Correct antenna placement in the lesion target (which was feasible in all cases despite the difficulty level raised by the challenging location) along with the reported technical efficacy rate provide preliminary evidence that the present concept is technically possible, functional, and producible and can act as a working model.

There are different devices for percutaneous needle guidance. These needle placement systems can be divided into two groups: (a) active guidance needle placement (mounted on patient, table, gantry, floor), and (b) passive guidance needle placement (additional feedback of needle position). The system used in the present study can be classified as a patient-mounted device that is positioned on the external skin surface of the patient during the procedure. In the literature, these devices have already been described and discussed; however, in all reported cases, navigation guidance and ablation were performed under deep sedation or general anesthesia (including jet ventilation). Similar to other studies, in the present case series, the treatment of malignant liver lesions with stereotactic navigation of microwave ablation was successful and well tolerated. One major difference of the present study is that all patients were treated under local anesthesia combined with intravenous analgesia, resulting, however, in no significant differences concerning the efficacy and safety rates. To our knowledge, the present study is the first one evaluating the performance of a patient-mounted device for navigation of tumor ablation under local anesthesia.

Limitations of the present study include the small number of participants and the lack of comparison to a group of patients undergoing alternative (i.e., guidance and ablation performed under deep sedation or general anesthesia) approaches. Furthermore, from an oncologic point of view, including in the same patient pool various oncologic substrates limits the validity of the survival rates; however, the present study aimed to primarily focus upon the feasibility and technical efficacy of performing percutaneous navigation under local anesthesia for computed tomography-guided microwave ablation of malignant liver lesions located in the hepatic dome.

## 5. Conclusions

In conclusion, the findings of the present study indicate that percutaneous navigation under local anesthesia is a safe and efficacious approach for computed tomography-guided microwave ablation of malignant liver lesions located in the hepatic dome. Large randomized controlled studies are warranted to observe treatment effectiveness and compare the results with those of other options.

## Figures and Tables

**Table 1:** Demographic and clinical data of the selected patients and lesion after MWA.

Demographics	Total Group
Patients (*n*)	10
Lesions (*n*)	16
Age (yrs)	60.60 ± 9.25
Gender (M/F)	8/2
Tumor type	HCC [2/10 (20%)], CLM [4/10 (40%)], GIST [2/10 (20%)] myeloid carcinoma of the thyroid gland [2/10 (20%)]
Tumor diameter (mm)	20.37 ± 7.29

Note: HCC: hepatocellular carcinoma, CLM: Colorectal liver metastasis, GIST: gastrointestinal stromal tumor.

## Data Availability

The data presented in this study are available on request from the corresponding author.
